# Integrative pan-cancer and clinical cytokine profiling reveals a pro-inflammatory cytokine index for colon cancer peritoneal metastasis and immunotherapy response

**DOI:** 10.3389/fimmu.2026.1977517

**Published:** 2026-10-26

**Authors:** Caihong Wu, Xin Li, Dan Wang, Dongmei Han, Hao Jin, Weishuai Liu

**Affiliations:** 1 Center for Precision Cancer Medicine & Translational Research, Tianjin Cancer Hospital Airport Hospital, Tianjin, China; 2 Pathology Department, Tianjin Cancer Hospital Airport Hospital, Tianjin, China; 3 Department of Technology Transfer, Tianjin Cancer Hospital Airport Hospital, Tianjin, China; 4 Center for Precision Oncology Testing and Translational Research, Tianjin Medical University Cancer Institute & Hospital, National Clinical Research Center for Cancer, Tianjin Key Laboratory of Digestive Cancer, Tianjin’s Clinical Research Center for Cancer, Tianjin, China

**Keywords:** biomarker, cytokines, immunotherapy, pan-cancer, prognosis, tumor microenvironment

## Abstract

**Background:**

Cytokines are central to tumor immunity, yet their landscape and clinical utility across cancer types remain incompletely characterized.

**Methods:**

We selected a representative cytokine panel covering Th1/Th2/Th17, pro-inflammatory, regulatory, and chemokine functions. Using TCGA pan-cancer data, we derived a transcriptomic cytokine index (gCCI) as the difference between pro-tumor and anti-tumor ssGSEA scores and evaluated its associations with prognosis, immune microenvironment, and immunotherapy response. We further validated a protein-level cytokine index (pCCI) using the same formula in an independent cohort of 183 cytokine profiling assays from colorectal cancer patients.

**Results:**

The gCCI effectively distinguished tumor from normal tissue in colorectal cancer and stratified survival in multiple cancer types. High gCCI was associated with activated inflammatory pathways by GSEA and showed cancer-type-specific correlations with MSI, TMB, and immune cell infiltration. Notably, low gCCI correlated with better immunotherapy response and prolonged survival in two independent cohorts. In protein-level validation, pCCI was significantly associated with T stage, N stage, M stage, and tumor location (all p < 0.05), with M1c (peritoneal metastasis) showing the highest pCCI levels among M substages. Dichotomized analysis by median pCCI further revealed that high pCCI was significantly enriched in colon cancer patients with M1c, whereas no such associations were observed in rectal cancer.

**Conclusions:**

The cytokine index, at both transcriptomic and protein levels, is associated with prognosis and immunotherapy response, with its utility being cancer-type and site-specific. Its particular relevance to colon cancer and peritoneal metastasis warrants further validation in prospective studies.

## 1 Introduction

Cytokines are small-molecule proteins that mediate intercellular communication, orchestrating the recruitment, activation, and function of various immune cell subsets within the immune system. In the tumor microenvironment (TME), they play dual roles—exerting either pro-tumor or anti-tumor effects depending on context, concentration, and cellular source (1). Pro-inflammatory cytokines such as interferon-gamma (IFN-γ) and interleukin-2 (IL-2) are generally associated with enhanced anti-tumor immunity, whereas immunosuppressive cytokines like interleukin-10 (IL-10) and transforming growth factor-beta (TGF-β) can foster an immune-excluded or tolerant microenvironment.

The TME plays a pivotal role in shaping anti-tumor immunity and determining the efficacy of immunotherapy (2). The immune checkpoint blockade (ICB), has revolutionized the landscape of cancer treatment, offering durable clinical responses and improved survival outcomes across a spectrum of malignancies (3). However, the clinical benefit of ICB remains limited to a subset of patients, with response rates varying significantly across different cancer types. Moreover, Existing predictive biomarkers such as PD-L1 expression, tumor mutational burden (TMB) and microsatellite instability (MSI) all have their own drawbacks (4–6). This heterogeneity underscores the urgent need for robust biomarkers to predict prognosis and therapeutic response, thereby enabling more precise patient stratification and personalized treatment strategies.

The role of some individual cytokines in cancer has been established, and intervention studies on tumor immunotherapy have become extensive. For instance, IL-2 promotes T-cell activation and has been used in cancer immunotherapy, while interleukin-6 (IL-6) and interleukin-8 (IL-8) are often associated with tumor progression and poor prognosis (7–9). These cytokines are generally exerted by the tumor or stromal cells in the TMB in an autocrine or paracrine (or both) manner (10). A systematic pan-cancer characterization of multiple cytokine combinations, especially covering their coding genes—integrated expression, genomic alterations, epigenetic regulation, and clinical outcomes—remains lacking. Moreover, the translation of cytokine-based signatures from transcriptomic discovery to protein-level clinical validation has been rarely attempted.

To address these gaps, we selected a representative panel of cytokines that spans the major axes of anti-tumor immunity. They collectively represent key T-cell differentiation axes and inflammatory/regulatory networks: Th1-type (IL-2, IFN-γ, IL-12p70), Th2-type (IL-4, IL-5), Th17-type (IL-17), pro-inflammatory (IL-1β, IL-6, TNF-α), regulatory (IL-10), and chemokine/interferon-related (IL-8, IFN-α) functions. We integrated large-scale pan-cancer genomic and transcriptomic data from The Cancer Genome Atlas (TCGA) with independent immunotherapy cohorts (GSE91061, IMvigor210) to construct a composite cytokine index (gCCI) reflecting the balance between pro-tumor and anti-tumor immune tone. We further validated its clinical relevance at the protein level using an in-house cohort of 183 cytokine profiling assays from colorectal cancer patients.

Our integrative approach revealed that gCCI stratifies survival across multiple cancer types and is associated with immune cell infiltration, inflammatory pathways, and immunotherapy response. Notably, protein-level validation uncovered a site-specific association in colorectal cancer (CRC): high pCCI was specifically enriched in colon cancer patients with peritoneal metastasis (M1c), while no such association was observed in rectal cancer, highlighting the biological heterogeneity within CRC. These findings position CCI as a potential biomarker for prognosis and immunotherapy response, with particular relevance to colon cancer and peritoneal dissemination-a clinically challenging metastasis pattern with limited therapeutic options.

## 2 Materials and methods

### 2.1 Data sources and cohorts selection

Transcriptomic data, clinical information, and mutation data for 33 cancer types were downloaded from TCGA database via the GDC data portal. Promoter methylation data (beta values) were retrieved from the TCGA Methylation450 array dataset. For external validation, two independent immunotherapy cohorts were used: GSE91061 (melanoma patients treated with anti-PD-1 therapy) and IMvigor210 (urothelial carcinoma patients treated with anti-PD-L1/CTLA-4 therapy). The response of immunotherapy was defined according to RECIST criteria.

A pilot cohort of clinical cytokine profiling was derived from peripheral blood samples of CRC patients who were diagnosed and treated at the Tianjin Cancer Hospital Airport Hospital from January 2025 to April 2026. We retrospectively recorded their clinicopathological data, including age, TNM, Stage, etc. All patients signed the informed consent form. This study was approved by the Ethics Committee of Tianjin Cancer Hospital Airport Hospital (Approval No. KY-2024-0049). Disease status (PD was determined by the treating physicians based on comprehensive clinical and radiological (primarily CT) assessments. Peripheral blood samples were collected from all patients prior to the initiation of any anti-tumor treatment (including surgery, chemotherapy, and immunotherapy).

### 2.2 Detection method of plasma cytokine levels

Plasma cytokine levels were quantified using flow cytometry-based multiplex immunoassay. All specimens were processed within 24 hours of collection and kept at 4 °C until analysis. Plasma was isolated from EDTA-K_2_ anticoagulated blood by centrifugation at 2,000 rpm for 10 minutes. Cytokine levels were measured using a flow cytometric twelve-cytokine assay kit (Uni-medica, Changsha, China). The lower limit of detection (LOD) for all cytokines was ≤ 2.44 pg/mL, as reported by the manufacturer. Values below the LOD were retained as reported by the assay software. Instrument calibration was performed using setup beads according to the manufacturer”s instructions, and kit-provided controls were included in each batch to monitor assay performance. Acquisition was carried out on a DxFLEX flow cytometer (Beckman Coulter), and data were analyzed using LEGENDplex software.

### 2.3 Copy number variation, mutation, and methylation analysis

Mutation data were visualized using the maftools R package, with mutation frequency calculated as the proportion of samples harboring at least one nonsynonymous mutation in each gene. For methylation analysis, promoter methylation beta values were extracted, and the absolute mean methylation difference (delta) between tumor and normal tissues was calculated.

### 2.4 Differential expression and prognostic analysis

Differential expression analysis between tumor and adjacent normal tissues was performed using the limma package in R, with |log2 fold change| > 1 and false discovery rate (FDR) < 0.05 considered statistically significant. For overall survival (OS) analysis, univariate Cox regression and Kaplan-Meier methods were applied using the survival and survminer R packages. Hazard ratios (HRs) with 95% confidence intervals (CIs) and log-rank p-values were calculated for each cytokine across cancer types with sufficient sample sizes.

### 2.5 Construction of the cell cytokine index

To capture the integrated effect of this functionally diverse cytokine panel, we defined two parallel indices at the transcriptomic and protein levels. The cytokines were classified into pro-tumor and anti-tumor groups based on their predominant functional roles in tumor immunity, consistent with the established type 1/type 2 cytokine paradigm (38). Pro-tumor cytokines (IL-1β, IL-6, IL-8, IL-10, IL-17A, IL-4, IL-5) have been reported to promote immunosuppression, angiogenesis, and epithelial-mesenchymal transition (EMT), while anti-tumor cytokines (IL-2, IFN-γ, TNF-α, IL-12, IFN-α) are canonical Th1-type effectors (39–41). For the gCCI (transcriptomic cytokine index), pro-tumor and anti-tumor cytokine-encoding gene sets were subjected to single-sample gene set enrichment analysis (ssGSEA) as implemented in the GSVA R package (version 1.46.0) (42). The ssGSEA method was run with default parameters: alpha = 0.25 and normalize = TRUE. The gCCI was calculated as: ssGSEA(pro-tumor genes) − ssGSEA(anti-tumor genes). For the pCCI (protein cytokine index), the same classification was applied to the in-house cytokine data, the mean concentration of pro-tumor cytokines was calculated for each sample, as was the mean concentration of anti-tumor cytokines. The pCCI was defined as: mean(pro-tumor cytokines) − mean(anti-tumor cytokines). Both indices were dichotomized by the median value for subsequent group comparisons.

### 2.6 Immune microenvironment and TMB/MSI analysis

Immune cell infiltration scores were estimated using the EPIC and CIBERSORT algorithm. TMB and MSI status data were obtained from previous TCGA pan-cancer studies. Radar plots were generated to visualize cancer-specific correlations between the gCCI and TMB/MSI.

### 2.7 Statistical analysis

All analyses used R (version 4.1.0); two-sided p < 0.05 was significant. Benjamini-Hochberg correction were applied to pan-cancer differential expression and GSEA analyses. Group comparisons used Chi-square or Fisher’s exact test, and continuous variables used Wilcoxon or Kruskal-Wallis tests. Interaction tests used logistic regression with an interaction term (pCCI × tumor location). In the clinical validation cohort, patients were categorized as M1c versus non-M1c (M0 + M1a), with Mx (M stage not further subclassified) cases excluded from the primary analysis and included in a sensitivity analysis. These stratified analyses were exploratory.

## 3 Results

### 3.1 Pan-cancer expression landscape and prognostic significance of a representative cytokine panel

To investigate the transcriptional landscape of a functionally representative set of cytokine-encoding genes—covering Th1, Th2, Th17, pro-inflammatory, regulatory, and interferon families—we systematically analyzed their expression patterns across 33 cancer types from TCGA. The expression of these cytokines varied markedly across different cancer types (**Figure 1A**). Specifically, IL-6 expression was significantly downregulated in BLCA, BRCA, KICH, KIRP, LIHC, LUAD, and LUSC. In contrast, IL-8 was significantly elevated in CESC, CHOL, COAD, ESCA, READ, and STAD. Notably, most interferon-alpha family genes (IFNA1–10 and IFNA14-17) exhibited coordinated downregulation in COAD, ESCA, READ, and UCEC, whereas IFNA13 showed no significant change in ESCA. Intriguingly, a substantial proportion of these cytokine genes were simultaneously downregulated in COAD, ESCA, READ, and UCEC, suggesting a potential common regulatory mechanism in these gastrointestinal malignancies.

**Figure 1:**
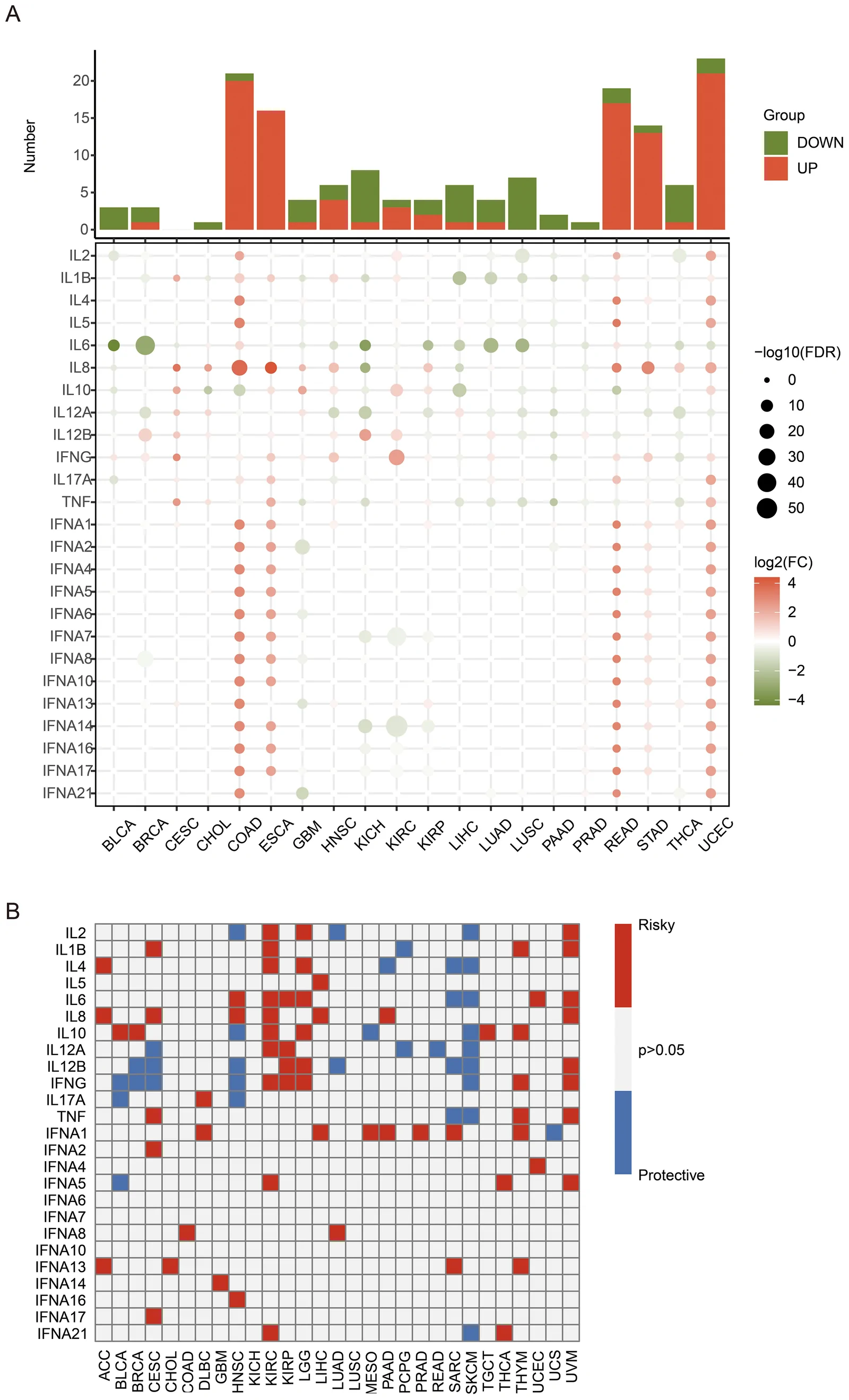
Expression and prognostic landscape of the selected cytokine-encoding genes across TCGA cancer types. **(A)** Expression of the cytokine coding genes in pan-cancer. **(B)** Impact of the cytokine coding genes expression on overall survival (OS).

We further assessed the prognostic value of each cytokine using Cox regression and Kaplan-Meier analyses. The same cytokine often exerted opposite prognostic effects across different cancer types (**Figure 1B**). For example, the high expression of IL-2 was associated with favorable OS in HNSC, LUAD, and SKCM, but correlated with poor prognosis in KIRC, LGG, and UVM. The overexpression of IL-8 was linked to adverse outcomes in ACC, LIHC, and PAAD. Conversely, the high expression of IL-12A expression was a favorable prognostic factor in CESC, READ, and SKCM. These results demonstrate that the expression and prognostic impact of cytokine genes are highly cancer-type specific.

### 3.2 Genomic and epigenetic alterations of the cytokine panel

We next examined the genomic alterations of this cytokine panel. CNV analysis revealed cancer-specific correlations with gene expression (**Figure 2A**). Notably, the CNV of IL-10 was negatively correlated with its expression in LIHC and LUAD (Spearman’s r < -0.2, p < 0.05), whereas the CNV of IL-12B showed positive correlations in HNSC, LUAD, and LUSC (r > 0.2, p < 0.05) (**Figure 2B**).

**Figure 2:**
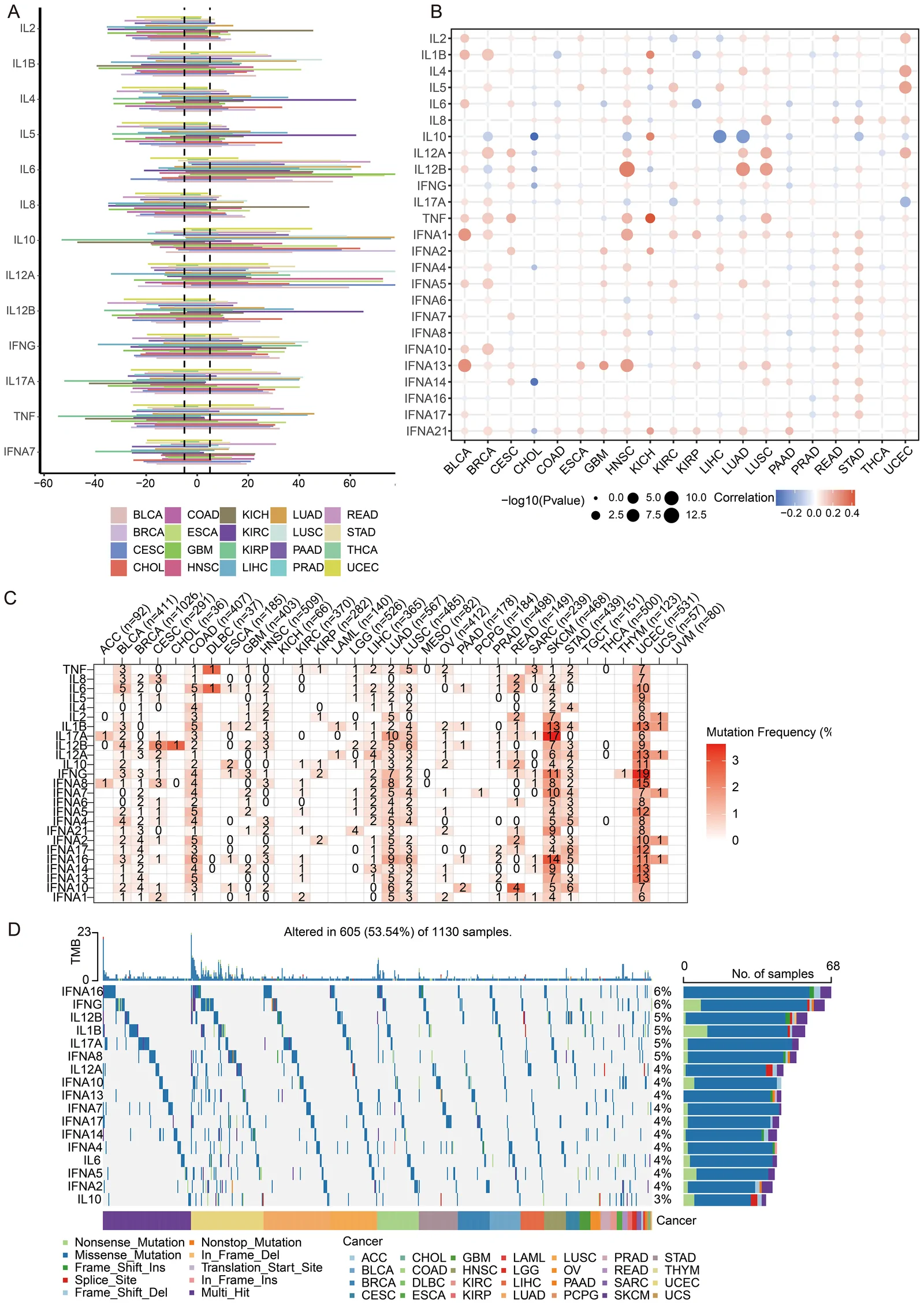
Genomic alterations of the cytokine genes in pan-cancer. **(A)** Copy number variation (CNV) rates of cytokine genes across cancer types. **(B)** Correlation between CNV rate and gene expression. **(C)** Mutation frequency of cytokine genes in pan-cancer. **(D)** Waterfall plot displaying the mutation landscape of cytokine genes across individual samples.

Mutation analysis indicated that over half of all samples harbored at least one mutation in these cytokine genes, and the mutation frequency was significantly heterogeneous among different genes and cancer types (**Figure 2C**). IFNA16 and IFNG had the highest mutation frequency (6% each), followed by IL12B, IL1B, IL17A, and IFNA8 (5% each) (**Figure 2D**). Missense mutations were the predominant alteration type. SKCM and UCEC showed the highest mutation burdens among all cancer types. The mutation events of these genes have a certain positive correlation with TMB, and are more concentrated in samples with high TMB.

Promoter methylation analysis showed cancer-specific patterns. The absolute mean methylation difference (delta) between tumor and normal tissues varied across cancers (**Figure 3A**). Notably, the promoter methylation of IL-10 correlated negatively with its expression in multiple cancers, while the methylation of IL-12B showed predominantly positive correlations with its expression, suggesting divergent epigenetic regulatory mechanisms (**Figure 3B**). Based on the previous mutation analysis, the mutation frequency of IL-10 in pan-cancer is relatively low, suggesting that its down-regulation of expression may be more influenced by epigenetic regulation rather than gene mutations.

**Figure 3:**
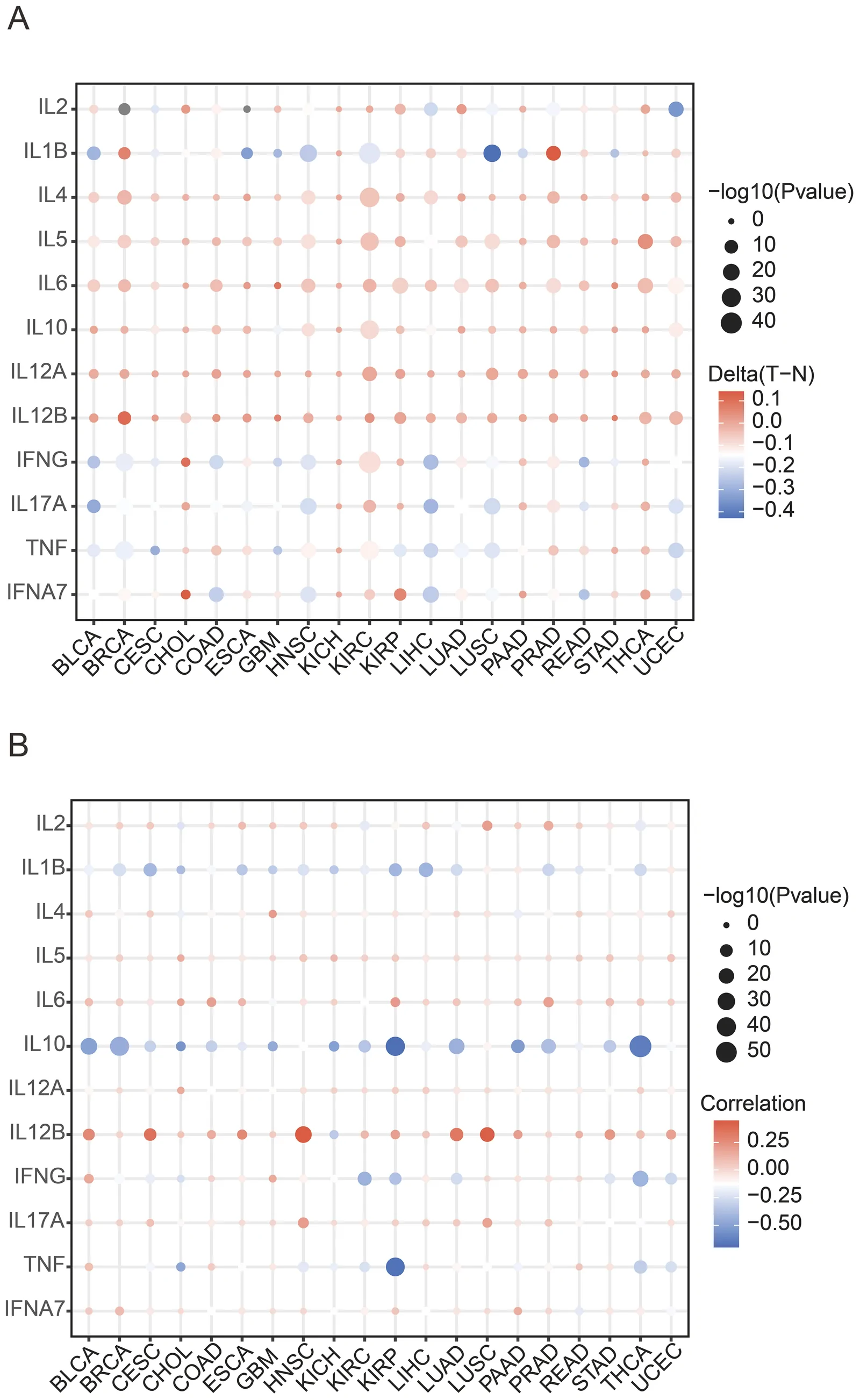
Epigenetic regulation of cytokine genes via promoter methylation. **(A)** Delta methylation between tumor and normal tissues for each cytokine gene, showing the magnitude of methylation change. **(B)** Correlation between promoter methylation level and gene expression across cancer types (Spearman’s correlation).

### 3.3 Construction and prognostic value of the transcriptomic cytokine index

To capture the integrated effect of this functionally diverse cytokine panel, we constructed a composite gCCI based on the expression levels of all the selected genes. The gCCI significantly distinguished tumor from normal tissue in multiple cancer types (**Figure 4A**). The gCCI was significantly higher in tumor tissues compared to adjacent normal tissues in ESCA, COAD, READ, STAD. The gCCI was significantly lower in tumor tissues compared to adjacent normal tissues in LIHC, KICH, BRCA, BLCA. The forest plot revealed significant cancer-type heterogeneity in the prognostic effects of gCCI: elevated gCCI was significantly associated with increased risk of mortality in multiple cancer types, including ACC (HR = 71.888, p = 0.002), CESC (HR = 23.142, p < 0.001), KIRC (HR = 9.568, p < 0.001), and LIHC (HR = 6.758, p = 0.008). In contrast, gCCI acted as a significant protective factor in thymic carcinoma (THYM), where higher gCCI was associated with reduced risk of mortality (HR = 0, 95% CI: 0–0.155, p = 0.022) (**Figure 4B**). Given the extreme HRs in some cancer types, we assessed model stability by performing Firth’s penalized likelihood analysis for sparse-event cases. Multivariable Cox regression adjusting for age and stage confirmed gCCI as an independent prognostic factor in several cancer types, including KIRC, HNSC, LIHC, ESCA, and SKCM (**Supplementary Table 1**).

**Figure 4:**
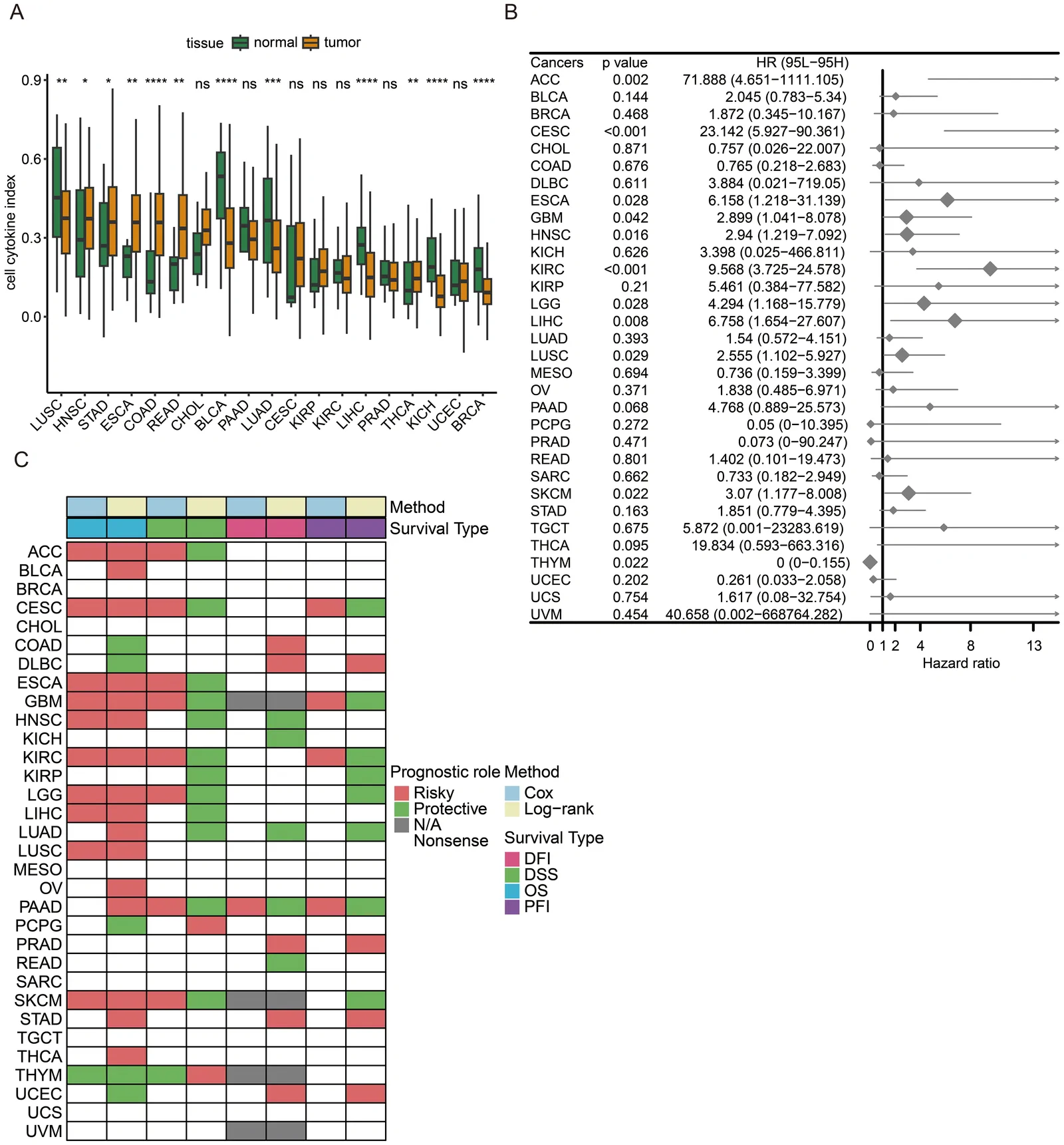
Construction and prognostic evaluation of the cell cytokine index (gCCI). **(A)** Comparison of gCCI between tumor and adjacent normal tissues across cancer types. **(B)** Forest plot showing the association of gCCI with overall survival risk across cancer types (Cox regression). **(C)** Pan-cancer prognostic heatmap summarizing the association of gCCI with multiple survival endpoints, including overall survival (OS), disease-specific survival (DSS), disease-free interval (DFI), and progression-free interval (PFI).

Motivated by the substantial heterogeneity observed in the forest plot, we further generated a pan-cancer prognostic heatmap to comprehensively characterize the role of gCCI across different survival endpoints, including OS, disease-specific survival (DSS), disease-free interval (DFI), and progression-free interval (PFI) (**Figure 4C**). Consistent with the HR > 1 findings from the forest plot, gCCI predominantly functioned as a risk factor in most cancer types, with stable adverse effects observed across multiple survival endpoints in ACC, CESC, KIRC, LGG, and others. Meanwhile, protective effects of gCCI were also noted in select cancer types such as COAD/READ, and KIRP. Notably, the protective role of gCCI in THYM was consistently validated across multiple survival outcomes (including OS and DSS), providing cross-method confirmation of the forest plot results.

Collectively, gCCI exhibits robust prognostic utility across pan-cancer settings, with highly cancer-type-specific effects: it serves as an adverse prognostic factor in the majority of tumors, while conferring a protective effect in specific malignancies, particularly THYM. These findings provide multi-dimensional evidence to support the translational potential of the inflammatory cytokine pathway in clinical practice.

### 3.4 Pathway enrichment and immune correlates of gCCI

We next investigated the biological pathways associated with the gCCI. GSEA revealed that samples with high gCCI were significantly enriched in immune-related pathways, including TNFA signaling via NFκB, inflammatory response, hypoxia, EMT, and KRAS signaling (FDR < 0.05, **Figure 5A**). Using the CIBERSORT and EPIC algorithm, we found that the gCCI positively correlated with cancer-associated fibroblasts (CAFs), CD4+ T cells, endothelial cells, and macrophages (**Figures 5B, C**). Across most cancer types, the cytokine index was broadly positively correlated with activated mast cells and neutrophils, while correlations with activated dendritic cells and eosinophils were weaker. These findings suggest that a high gCCI may be associated with a protumoral inflammatory milieu, although these correlations may partly reflect variation in cellular composition. The gCCI showed cancer-specific correlations with TMB and MSI (**Figures 5D, E**). Notably, it was negatively correlated with MSI status in ACC, BLCA, COAD, HNSC, LUAD, and LUSC, but positively correlated in LIHC. The positive correlations were observed bewteen gCCI and TMB in COAD and KIRC.

**Figure 5:**
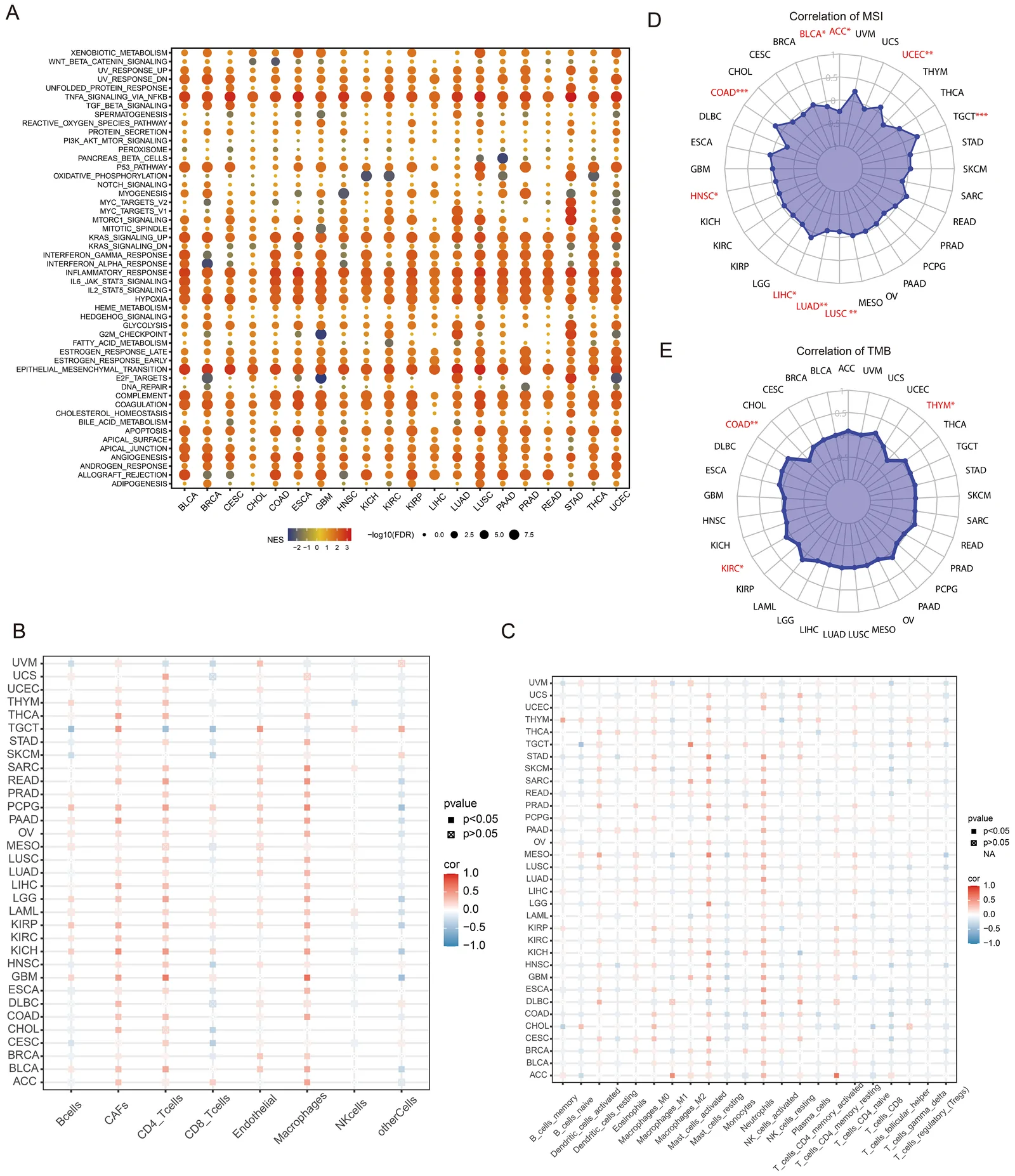
Immune molecular characteristics associated with gCCI. **(A)** Gene set enrichment analysis (GSEA) revealing pathways enriched in high- vs low-gCCI samples. **(B)** Correlation between gCCI and immune cell infiltration estimated by EPIC. **(C)** Correlation between gCCI and immune cell infiltration estimated by CIBERSORT. **(D)**Radar chart showing correlations between gCCI and microsatellite instability (MSI) score. **(E)** Radar chart showing correlations between gCCI and tumor mutational burden (TMB).

### 3.5 Association of gCCI with immunotherapy response

Given the central role of cytokines in immune regulation, we examined the association of gCCI with ICB response. In the GSE91061 cohort, patients with low gCCI showed significantly higher response rates and longer overall survival compared to those with high gCCI (p < 0.05, **Figures 6A, B**).

**Figure 6:**
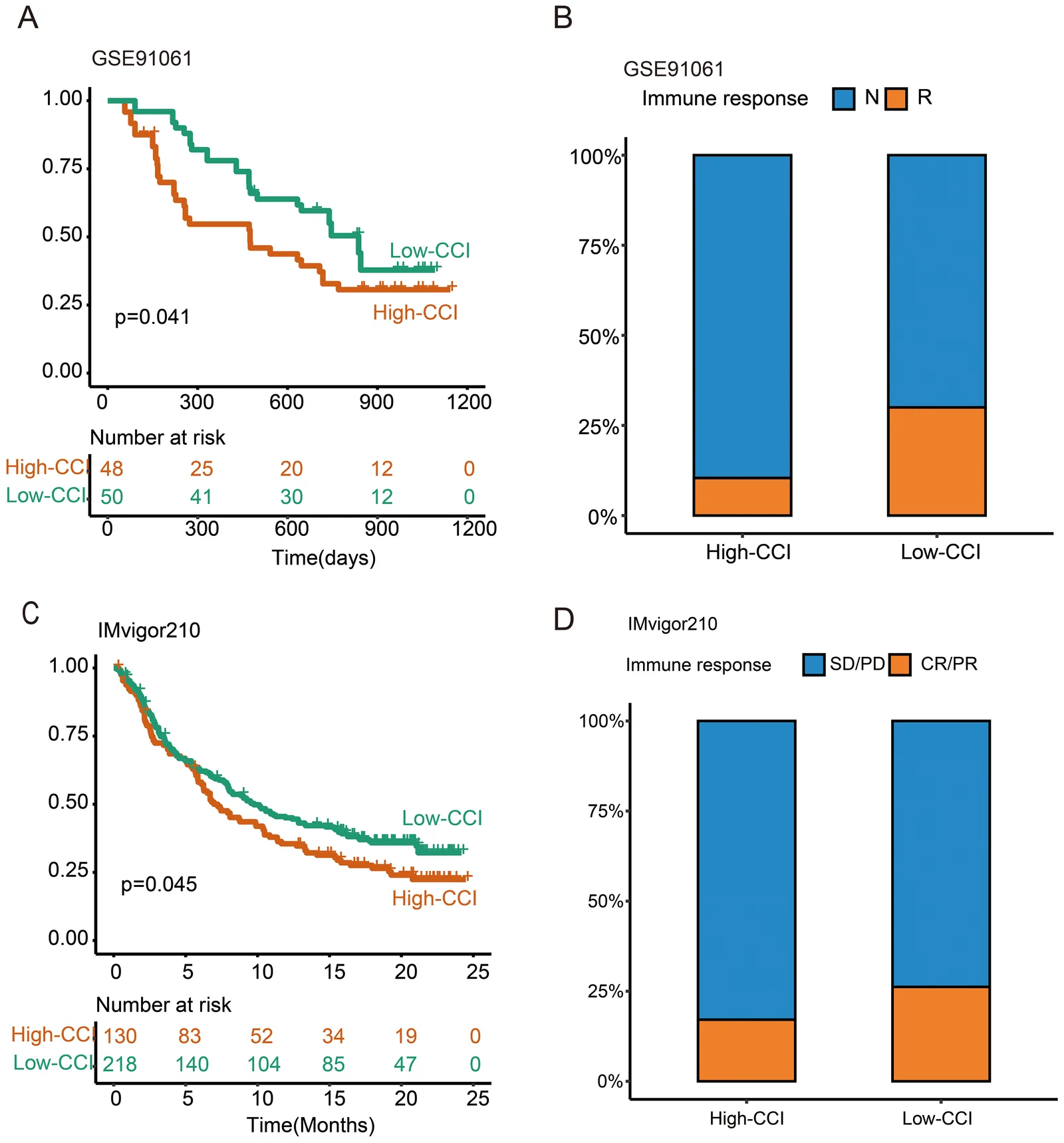
Association of gCCI for immune checkpoint blockade (ICB) response. **(A)** Kaplan-Meier curves comparing overall survival between low- and high-gCCI groups in the GSE91061 cohort. **(B)** Distribution of ICB response across low- and high-gCCI groups in the GSE91061 cohort. **(C)** Kaplan-Meier curves comparing overall survival between low- and high-gCCI groups in the IMvigor210 cohort. **(D)** Distribution of ICB response across low-and high-gCCI groups in the IMvigor210 cohort. R, responser; N, non-responser; CR, complete response; PR, partial response; SD, stable disease; PD, progressive disease.

These findings were validated in the IMvigor210 cohort. The patients with low gCCI again demonstrated better clinical outcomes, with higher rates of Complete Response (CR) and Partial Response (PR) (**Figures 6C, D**). Collectively, these results indicate that a lower gCCI is associated with favorable ICB response across independent cohorts and therapeutic regimens.

### 3.6 Independent validation of the protein-level cytokine index in a plasma cohort

To validate the clinical relevance of gCCI at the protein level, we calculated pCCI in an independent plasma cohort of 183 cytokine profiling assays from CRC patients and evaluated its associations with clinicopathological features. As shown in **Table 1**, pCCI levels differed significantly across T stage (p = 0.004), N stage (p = 0.011), M stage (p < 0.0001), and tumor location (p = 0.0082), but not across age, sex, or clinical stage (p > 0.05). Pairwise comparisons further revealed stage-specific patterns (**Figures 7A–C**): pCCI was significantly higher in T4 than in T3; for N stage, it exhibited a non-monotonic U-shaped pattern (N0 > N1 < N2); and most strikingly, M1c showed the highest pCCI levels among all M substages, whereas M1a did not differ from M0.

**Table 1:** Clinicopathological characteristics of colorectal cancer patients stratified by pCCI level.

Characteristics	n	pCCI value	*P*-value
Age		Median (IQR)	
≤60	125	1.254(0.134 – 2.500)	0.060
>60	58	0.634 (-0.294 – 1.801)	
Gender		Median (IQR)	
Male	98	1.136 (-0.006 – 2.048)	0.526
Female	85	1.082 (-0.184 – 2.735)	
Clinical stage		Median (IQR)	
I–II	21	1.507 (0.488 – 3.288)	0.348
III	64	1.022 (-0.293 – 2.411)	
IV	98	0.988 (-0.216 – 2.220)	
Tumor (T stage)		Median (IQR)	
T1-T2	11	1.368 (0.789 – 2.886)	0.004
T3	131	0.924 (-0.265 – 1.960)	
T4	41	2.304 (0.340 – 4.169)	
Lymph node metastasis (N stage)		Median (IQR)	
N0	25	1.368 (0.173 – 2.732)	0.011
N1	83	0.688 (-0.345 – 1.601)	
N2	75	1.713 (0.169 – 2.982)	
Distant metastasis (M stage)		Median (IQR)	
M0	85	1.140 (0.053 – 2.540)	<0.0001
M1a	24	0.310 (-0.595 – 2.161)	
M1c	22	3.147 (1.519 – 10.330)	
Mx	52	0.738 (-0.281 – 1.765)	
Tumor location		Median (IQR)	
Colon	72	1.614 (0.063 – 3.390)	0.0082
Rectum	111	0.924 (-0.205 – 1.960)	

p values were calculated using Wilcoxon rank-sum test (for two groups) or Kruskal-Wallis test (for three or more groups). Mx indicates M stage not further subclassified.

**Figure 7:**
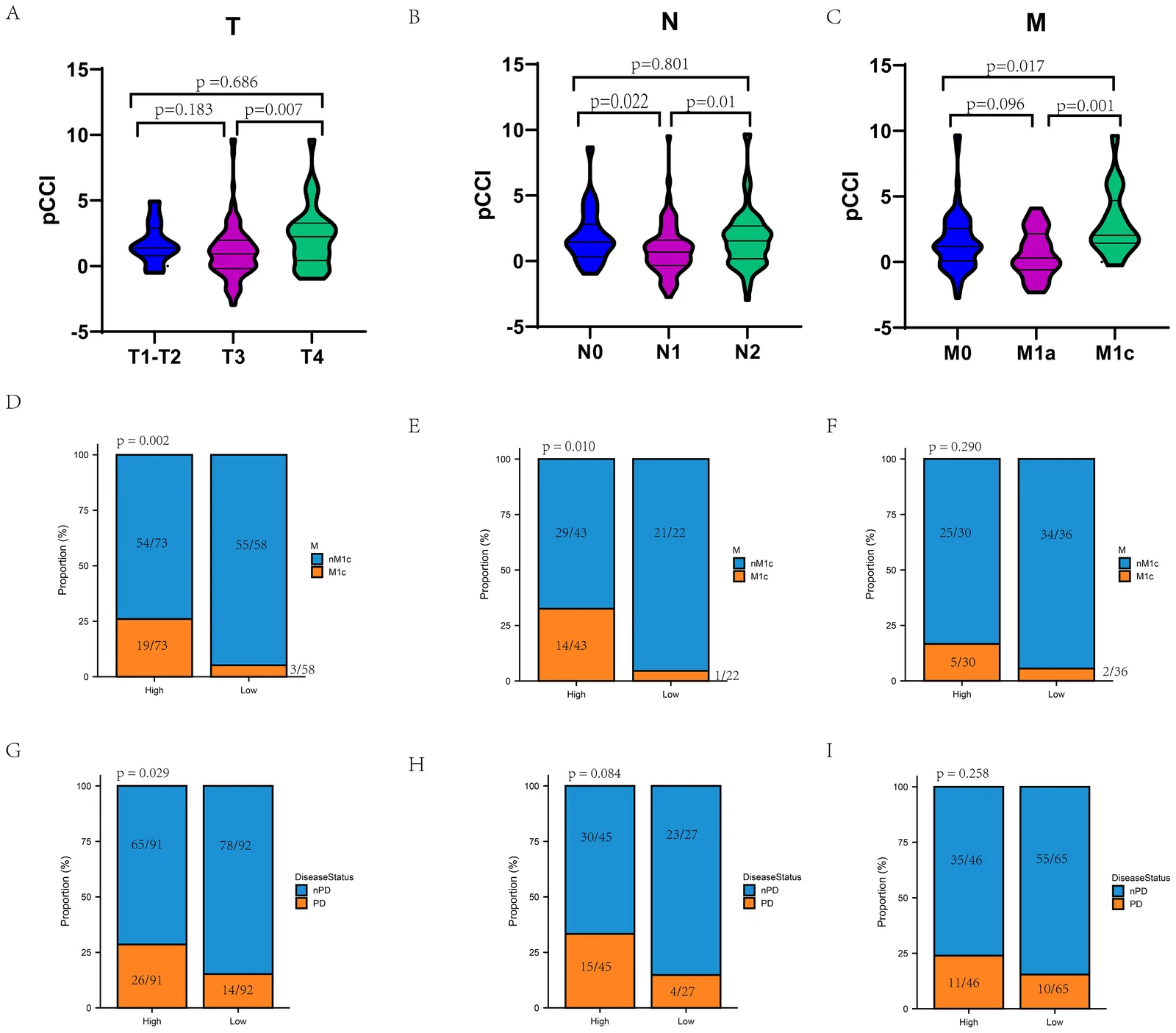
Association of pCCI with clinicopathological features, peritoneal metastasis, and disease progression. **(A-C)** Distribution of pCCI across clinical substages. Violin plots showing pCCI distribution across **(A)** T stage, **(B)** N stage, and **(C)** M stage subgroups. **(D-F)** Association with peritoneal metastasis (M1c). Bar plots showing the proportion of M1cand non-M1c across high- and low-pCCI groups in **(D)** total cohort, **(E)** colon cancer, and **(F)** rectal cancer. **(G-I)** Association with disease progression (PD vs non-PD). Bar plots showing the proportion of PD across high- and low-pCCI groups in **(G)** total cohort, **(H)** colon cancer, and **(I)** rectal cancer. PD, progressive disease.

To directly evaluate the specificity of pCCI for peritoneal metastasis, we reclassified M stage into M1c versus non-M1c. Because 52 patients had Mx and could not be confirmed as having or not having peritoneal metastasis, they were excluded from the primary analysis, leaving 131 patients with confirmed M0, M1a, or M1c status (65 colon, 66 rectum). In this Mx-excluded cohort, the proportion of M1c was significantly higher in the high-pCCI group in the total cohort (p = 0.002), predominantly observed in colon cancer (p = 0.01), with no significant association in rectal cancer (p = 0.29) (**Figures 7D–F**). The full-cohort analysis including Mx cases yielded consistent results (**Supplementary Figure 1**). A sensitivity analysis using z-score standardized concentrations showed moderate correlation with the original pCCI (Spearman rho = 0.651) but attenuated the M1c association (**Supplementary Figure 2**). We therefore retained the original pCCI as the primary analysis.

We further examined the association between pCCI and disease status (Progressive Disease, PD vs non-PD) (**Figures 7G–I**). In the total cohort, high pCCI was associated with a higher proportion of PD (28.6% vs 15.2%, p = 0.029). Subgroup analysis again showed that this association was only a non-significant trend (33.3% vs 14.8%, p = 0.084) in the colon cancer, and no such trend was observed in rectal cancer (23.9% vs 15.4%, p = 0.258). Collectively, these preliminary findings suggest that pCCI is associated with peritoneal metastasis in colon cancer, although this site-predominant pattern requires independent validation in larger cohorts.

## 4 Conclusion

In summary, our integrative approach—from pan-cancer genomic analysis to protein-level validation—demonstrates that CCI is a cancer-type-specific biomarker associated with prognosis and immunotherapy response. Its site-predominant association with colon cancer and peritoneal metastasis underscores the importance of stratified biomarker development within colorectal cancer. These findings warrant prospective evaluation of CCI as a potential tool for risk stratification and immunotherapy guidance.

## 5 Discussion

Tumor immunotherapy has ushered in a new era in the field of tumor treatment. Its therapeutic effect is closely related to the immune function status of the host. Novel biomarkers that reflect the integrated immune status of the host are urgently needed (11). Cytokines serve as critical mediators of intercellular communication, orchestrating the recruitment, activation, and function of various immune cell subsets in the TME. Despite growing evidence linking individual cytokines to cancer outcomes, a comprehensive cytokine index that integrates multiple cytokines to predict both prognosis and immunotherapy response across cancers has not been well established. Previous studies have often focused on single cytokines or small panels within specific cancer types, limiting the generalizability and clinical utility of the findings (12). Moreover, the interplay between systemic cytokine levels and the broader molecular and immunological context of the TME warrants further investigation.

To our knowledge, this is the first pan-cancer study to integrate expression, CNV, mutation, and methylation data for selected cytokine genes. The coordinated downregulation of multiple interferons in COAD/ESCA/READ/UCEC suggests a potential epigenetic silencing mechanism that warrants further investigation. This coordinated downregulation is intriguing, as it implies that gastrointestinal cancers may collectively suppress cytokine-mediated immune surveillance as an early event in tumorigenesis (13). The observation that the same cytokine exerted opposite prognostic effects across different cancer types further underscores the context-dependent nature of cytokine biology. IL-2, a canonical T-cell growth factor, was associated with favorable survival in HNSC, LUAD, and SKCM, yet predicted poor prognosis in KIRC, LGG, and UVM. This dichotomy may reflect the dual role of IL-2 in both activating effector T cells and expanding immunosuppressive regulatory T cells (Tregs), with the net effect depending on the composition of the tumor-infiltrating lymphocyte pool and the local TME (14–16). These findings collectively support the fact that cytokine genes exhibit highly cancer type-specific expression patterns and prognostic effects.

The composite CCI effectively captured the integrated immune tone across the selected cytokine panel and demonstrated robust prognostic utility across multiple cancer types. Elevated gCCI was associated with increased mortality risk in ACC, CESC, KIRC, LIHC, and several other malignancies, supporting the notion that a pro-inflammatory, immunosuppressive cytokine milieu promotes tumor progression and poor clinical outcomes (17). Notably, gCCI exhibited a striking protective effect in THYM, where higher gCCI was associated with reduced mortality risk. This paradoxical finding may be explained by the unique immunobiology of THYM—a tumor originating from the thymic epithelium, which plays a central role in T-cell development and selection (18). In THYM, elevated cytokine signaling may actually promote immune-mediated tumor control rather than immune evasion, highlighting the critical importance of tissue context in interpreting cytokine-based biomarkers (19). This cancer-type specificity was further validated across multiple survival endpoints (OS, DSS, DFI, PFI), reinforcing the conclusion that gCCI’s prognostic direction is not fixed but rather shaped by the underlying tumor-immune ecosystem.

GSEA revealed that high-gCCI samples were significantly enriched in pro-tumorigenic pathways, including TNFA signaling via NFκB, inflammatory response, hypoxia, EMT, and KRAS signaling. This enrichment pattern provides mechanistic support for gCCI as a reflection of an active, protumoral inflammatory state—one that fosters tumor invasion (EMT), metabolic adaptation (hypoxia), and sustained inflammatory signaling (NFκB) (20, 21). The positive correlation between CCI and CAFs/Macrophages is consistent with a protumoral inflammatory milieu, where these cells secrete cytokines that further drive immune evasion (22). Notably, CCI was negatively correlated with MSI in COAD. This is consistent with the known immunogenicity of MSI-high tumors and aligns with our observation that low gCCI correlates with better ICB response. Mechanistically, low gCCI—indicating dominance of anti-tumor over immunosuppressive cytokines—favors T-cell activation and ICB response (23). This signal was consistent across cancer types and therapeutic regimens, and unlike tissue-based markers (PD-L1, TMB, MSI), CCI captures systemic immune tone via peripheral profiling, potentially complementing existing biomarkers by reflecting both local TME and host immunity.

The translation of CCI from transcriptomic discovery to protein-level clinical validation represents a critical step toward clinical applicability. We calculated the pCCI using the same algorithm and verified its association with clinicopathological features. Especially, subgroup analysis revealed marked site-specific heterogeneity. Colon and rectal cancers differ substantially despite sharing the “colorectal” designation. They diverge in embryonic origin (midgut vs hindgut), molecular pathways, metastatic routes, and immune microenvironments (24–26). Peritoneal metastasis (M1c) is a particularly challenging complication of colon cancer, associated with poor prognosis, limited treatment options, and difficulty in early detection (27). Intestinal inflammation is a known risk factor for colorectal cancer. In a local inflammatory environment, cytokines play a role in the progression of colorectal cancer through immunosuppression and regulation of the tumor microenvironment. In particular, unresolved inflammation creates a microenvironment conducive to the transformation of immune cells and the growth of cancer cells (28). The balance between pro-inflammatory and anti-inflammatory factors determines the tumor response. In colorectal cancer, sustained activation of pro-inflammatory pathways exposes the TME to inflammatory cytokines. This inhibits anti-tumor immunity, induces EMT, and promotes tumor growth, ultimately driving treatment resistance and metastatic progression (29). Peritoneal metastasis reflects widespread tumor dissemination and generally indicates an advanced disease state in colorectal cancer (30). Cytokines mediate tumor microenvironment communication and promote widespread tumor spread (31). For instance, IL-6 and TNF-α synergistically activate STAT3 and NF-κB to promote colorectal cancer cell growth (32, 33), while IL-8 serves as a key predictor of overall survival in metastatic colorectal cancer (34). Since pro-inflammatory cytokines constitute the pro-tumor arm of pCCI, it is mechanistically plausible that high pCCI reflects an inflammatory milieu predisposing colon cancer to peritoneal metastasis. The lack of pCCI association in rectal cancer further supports this interpretation. Rectal cancer metastasis is predominantly hematogenous, driven by vascular invasion and angiogenesis pathways (e.g., VEGF, EGFR) rather than inflammatory peritoneal seeding. Indeed, peritoneal metastasis is significantly less frequent in rectal cancer (8.8%) compared to right-sided colon cancer (34.7%) (35), and the rectum’s venous drainage—partially via the inferior rectal vein into the inferior vena cava—favors lung over liver as the first site of hematogenous dissemination (25). Thus, pCCI—as an index of systemic inflammatory/immune balance—would not be expected to correlate strongly with clinical features in rectal cancer. Far from being a limitation, this site-specific pattern validates the biological specificity of pCCI and positions it as a potential tool for colon cancer peritoneal metastasis risk stratification, rather than a generic colorectal cancer marker.

This discovery has potential clinical translational value. Peritoneal metastasis is one of the most intractable metastasis patterns of colon cancer. It has an insidious onset, a very poor prognosis, and is difficult to be detected early by existing imaging methods (27). As a non-invasive indicator based on peripheral blood cytokines, pCCI is expected to be used for the early identification and risk stratification of high-risk populations with peritoneal metastasis of colon cancer, guiding more close imaging follow-up or preventive intervention strategies such as intraperitoneal perfusion chemotherapy. Combining the reported targeted NF-κB in colon cancer cells to promote the anti-tumor M1-like macrophage phenotype and inhibit peritoneal metastasis, the CCI index we constructed is helpful for discovering new therapeutic opportunities in anti-tumor treatment (36). Tumor regression after ICB is usually associated with immune-related adverse events (irAEs), marked by inflammation in non-cancerous tissues. The CCI we have established can help identify a balanced state between anti-tumor and anti-autoimmunity in the body, so as to enhance anti-tumor immunity while better managing irAE (37).

Several limitations should be acknowledged. First, The pCCI was calculated from absolute cytokine concentrations, which may be influenced by the differing magnitudes of individual cytokines. Standardization attenuated the M1c association, suggesting that the signal may be partially driven by high-abundance cytokines such as IL-6 and IL-8. This limitation should be addressed in future studies with larger cohorts. Second, we were unable to perform stable multivariable adjustment due to severe collinearity between pCCI and N stage and complete separation in T/N subgroups. The association between pCCI and M1c should therefore be considered preliminary, and confounding by overall disease burden cannot be fully excluded. Independent validation in larger cohorts is needed. Third, while our findings reveal an association between pCCI and peritoneal metastasis, this study does not establish a causal role for CCI. Functional studies including *in vitro* adhesion assays and *in vivo* peritoneal dissemination models are required to determine whether CCI directly contributes to peritoneal dissemination. Finally, since GSE91061 and IMvigor210 are single-arm cohorts without untreated controls, our findings demonstrate an association between gCCI and immunotherapy response rather than formal predictive utility. Future studies with treatment-by-biomarker interaction analyses or randomized cohorts are needed to establish whether gCCI predicts immunotherapy benefit. In addition, the correlations between gCCI and immune cell infiltration may partly reflect variation in cellular composition rather than an independent cytokine signaling state. Due to technical constraints, partial correlation analysis adjusting for tumor purity could not be performed, and future studies are needed to confirm these associations.

Despite these limitations, our findings suggest a site-predominant association between pCCI and disease progression in colon cancer, although the colon cancer subgroup did not reach statistical significance. The fact that this association remained detectable despite the inherent challenges of matching biomarker measurements with clinical assessments in a real-world setting suggests that pCCI may reflect a relatively stable biological state associated with tumor progression, rather than an instantaneous response to a single treatment event. This warrants further validation in larger prospective cohorts.

## Data Availability

Publicly available datasets analyzed in this study can be found here: The Cancer Genome Atlas (TCGA) pan-cancer transcriptomic data (https://portal.gdc.cancer.gov/); GSE91061 (https://www.ncbi.nlm.nih.gov/geo/query/acc.cgi?acc=GSE91061); IMvigor210 (http://researchpub.gene.com/IMvigor210CoreBiologies/). The in-house cytokine profiling data are not publicly available due to patient privacy restrictions but are available from the corresponding author upon reasonable request.
