# Supplementary material for: Integrative pan-cancer and clinical cytokine profiling reveals a pro-inflammatory cytokine index for colon cancer peritoneal metastasis and immunotherapy response
Source: Front Immunol. 2026 Oct 26;17:1977517. doi: 10.3389/fimmu.2026.1977517 (PMC13746216; doi:10.3389/fimmu.2026.1977517)
Supplement: Supplementary file 1 [file DataSheet1.pdf]

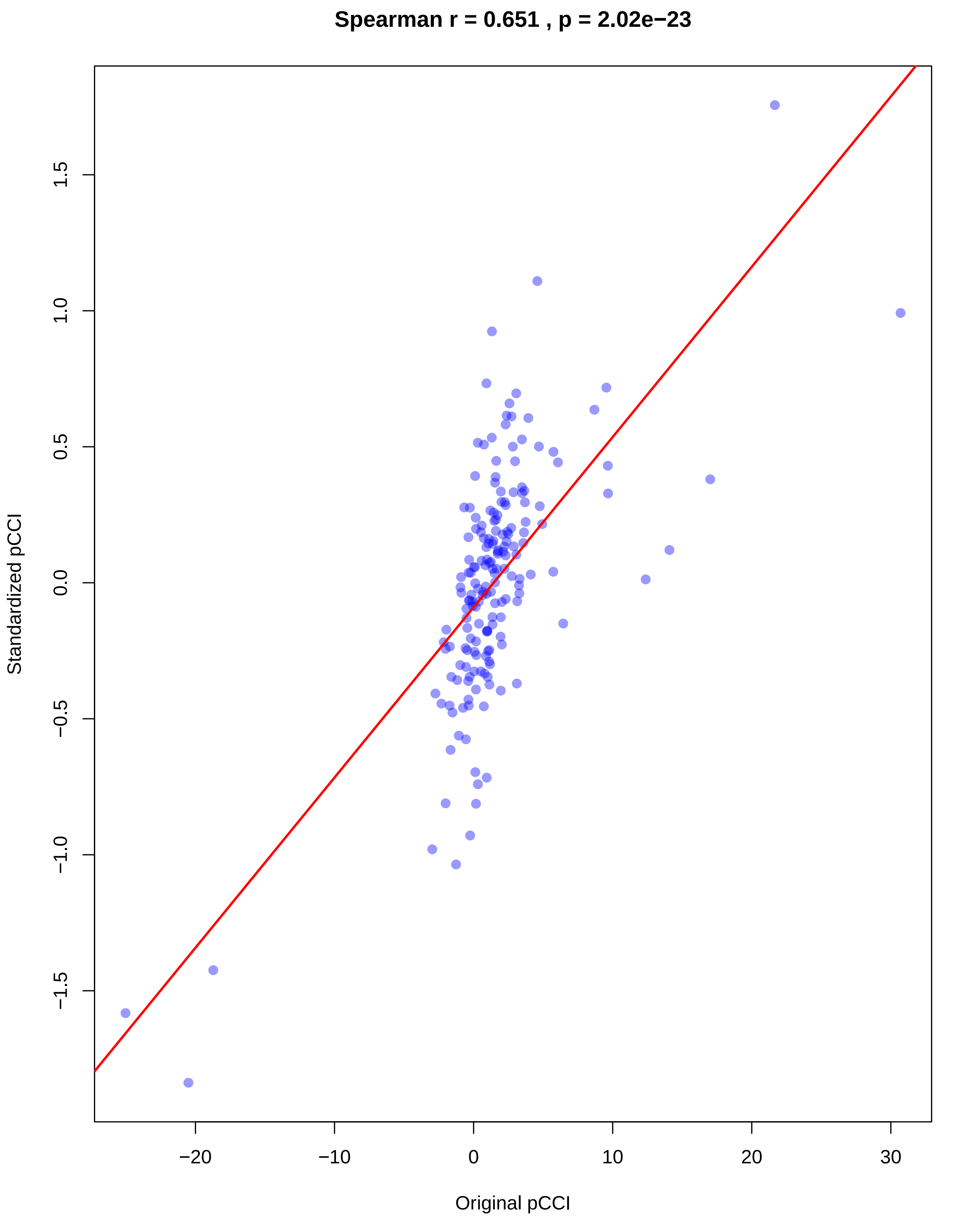
Spearman r = 0.651 , p = 2.02e−23
Standardized pCCI
Original pCCI
1.5
1.0
0.5
0.0
−0.5
−1.0
−1.5
−20
−10
0
10
20
30
