# Supplementary material for: Integrative pan-cancer and clinical cytokine profiling reveals a pro-inflammatory cytokine index for colon cancer peritoneal metastasis and immunotherapy response
Source: Front Immunol. 2026 Oct 26;17:1977517. doi: 10.3389/fimmu.2026.1977517 (PMC13746216; doi:10.3389/fimmu.2026.1977517)
Supplement: Supplementary file 2 [file Table1.docx]

**Supplementary Table 1 Sample sizes, event counts, and Cox regression results for gCCI across cancer types.**

| **Cancer type** | **Samples** | **events** | **Adjusted HR** | **Adjusted p** | **Firth HR** | **Firth p** | **Note** |
| --- | --- | --- | --- | --- | --- | --- | --- |
| ACC | 79 | 28 | 12.189 | 0.059 | — | — |  |
| BLCA | 407 | 178 | 1.851 | 0.213 | — | — |  |
| BRCA | 1101 | 154 | 3.446 | 0.164 | — | — |  |
| CESC | 306 | 72 | — | — | — | — | Not adjusted (non-convergence) |
| CHOL | 36 | 18 | 0.789 | 0.910 | — | — |  |
| COAD | 450 | 101 | 0.867 | 0.831 | — | — |  |
| ESCA | 185 | 78 | 6.989 | 0.047 | — | — |  |
| GBM | 166 | 133 | — | — | — | — | Not adjusted (non-convergence) |
| HNSC | 521 | 221 | 3.128 | 0.023 | — | — |  |
| KIRC | 534 | 175 | 7.084 | <0.001 | — | — |  |
| KIRP | 290 | 44 | 2.674 | 0.477 | — | — |  |
| LGG | 528 | 133 | — | — | — | — | Not adjusted (non-convergence) |
| LIHC | 372 | 130 | 6.099 | 0.027 | — | — |  |
| LUAD | 508 | 183 | 1.134 | 0.816 | — | — |  |
| LUSC | 495 | 212 | 2.314 | 0.054 | — | — |  |
| MESO | 86 | 73 | 0.424 | 0.312 | — | — |  |
| OV | 307 | 185 | — | — | — | — | Not adjusted (non-convergence) |
| PAAD | 179 | 93 | 3.911 | 0.150 | — | — |  |
| PRAD | 498 | 10 | — | — | — | — | Not adjusted (non-convergence) |
| READ | 160 | 24 | 0.475 | 0.625 | — | — |  |
| SARC | 263 | 99 | — | — | — | — | Not adjusted (non-convergence) |
| SKCM | 457 | 215 | 3.407 | 0.026 | — | — |  |
| STAD | 409 | 157 | 0.987 | 0.979 | — | — |  |
| THCA | 513 | 16 | 0.494 | 0.723 | — | — |  |
| UCEC | 532 | 88 | — | — | — | — | Not adjusted (non-convergence) |
| UCS | 57 | 35 | — | — | — | — | Not adjusted (non-convergence) |
| UVM | 80 | 23 | 4438.917 | 0.101 | — | — | Unstable estimate |
| DLBC | 48 | 9 | — | — | 6.234 | 0.496 | Sparse, Firth |
| KICH | 65 | 9 | — | — | 7.085 | 0.435 | Sparse, Firth |
| PCPG | 184 | 8 | — | — | 0.058 | 0.315 | Sparse, Firth |
| TGCT | 139 | 4 | — | — | 5.740 | 0.686 | Sparse, Firth |
| THYM | 119 | 9 | — | — | 0.000 | 0.026 | Sparse, Firth |

Adjusted HR: hazard ratio from multivariable Cox regression adjusting for age and stage. Firth HR: hazard ratio from Firth's penalized likelihood analysis for cancer types with fewer than 10 events. 'Not adjusted (non-convergence)': multivariable model did not converge due to sparse events. 'Unstable estimate': extreme HR with wide confidence interval. '—': not applicable
